# Mapping dominant annual land cover from 2009 to 2013 across Victoria, Australia using satellite imagery

**DOI:** 10.1038/sdata.2015.69

**Published:** 2015-11-24

**Authors:** Kathryn Sheffield, Elizabeth Morse-McNabb, Rob Clark, Susan Robson, Hayden Lewis

**Affiliations:** 1 Agriculture Research, Victorian Department of Economic Development, Jobs, Transport and Resources, Parkville, Victoria 3053, Australia; 2 Agriculture Research, Victorian Department of Economic Development, Jobs, Transport and Resources, Epsom, Victoria 3551, Australia; 3 Agriculture Research, Victorian Department of Economic Development, Jobs, Transport and Resources, Horsham, Victoria 3400, Australia; 4 Agriculture Research, Victorian Department of Economic Development, Jobs, Transport and Resources, Tatura, Victoria 3616, Australia

**Keywords:** Agriculture, Geography, Research data

## Abstract

There is a demand for regularly updated, broad-scale, accurate land cover information in Victoria from multiple stakeholders. This paper documents the methods used to generate an annual dominant land cover (DLC) map for Victoria, Australia from 2009 to 2013. Vegetation phenology parameters derived from an annual time series of the Moderate Resolution Imaging Spectroradiometer Vegetation Indices 16-day 250 m (MOD13Q1) product were used to generate annual DLC maps, using a three-tiered hierarchical classification scheme. Classification accuracy at the broadest (primary) class level was over 91% for all years, while it ranged from 72 to 81% at the secondary class level. The most detailed class level (tertiary) had accuracy levels ranging from 61 to 68%. The approach used was able to accommodate variable climatic conditions, which had substantial impacts on vegetation growth patterns and agricultural production across the state between both regions and years. The production of an annual dataset with complete spatial coverage for Victoria provides a reliable base data set with an accuracy that is fit-for-purpose for many applications.

## Background & Summary

Land cover describes the earth’s surface in terms of its physical cover such as grass, forest, impervious surfaces and water^1^. Regularly updated, accurate land cover information is important for a range of purposes including natural resource management, land cover change monitoring, agricultural research, infrastructure planning, fire management, ecological and hydrological modelling, and government policy development^2–7^. The production of an annual dataset facilitates the examination of land cover changes over time, inter-annual trends such as agricultural cropping rotation patterns, and responses of land cover to seasonal and annual climatic conditions. This study developed a method to produce an annual dominant land cover (DLC) map for the entire state of Victoria, Australia. Prior to this work existing approaches to land cover classification in Victoria were either focused on smaller areas, undertaken for specific purposes, recorded land use or enterprise rather than land cover information, or were not updated regularly^2,7–9^. Producing a regularly updated map with complete spatial coverage of Victoria and consistent land cover classes fills a data gap and supplies base data for a number of operational and research projects.

**Figure 1:**
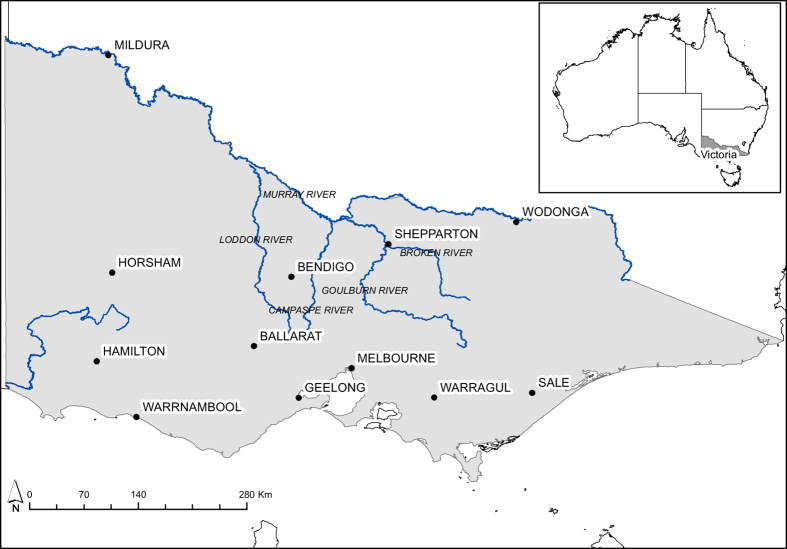
Location map of Victoria, Australia.

Victoria is located in south-east Australia (Fig. 1) and covers an area of over 23 million km^2^. More than half of the land (55%) is used for primary production with major enterprises including dryland cropping, irrigated agriculture, horticulture, dairy production, and livestock grazing. Approximately 38% of the state is public (government owned)land. More than 70% of the Victorian population of over 5.3 million live in the state’s capital, Melbourne. The climate varies from semi-arid in the north-west, alpine areas in the east and a more temperate climate in the south. Annual rainfall is higher in southern areas, with long-term annual averages across the state varying from less than 300 mm to more than 2,500 mm. Agricultural regions experience varying average annual rainfall from 280 mm in Mildura, 460 mm in Horsham and 1,022 mm in Warragul. The Murray River forms the northern border of the state.

This paper outlines a process used to produce a validated land cover map from 2009 to 2013 that provides a source of broad land cover information, with complete spatial coverage of the state of Victoria and which is updated on an annual basis. The land cover maps are intended as a source of broad land cover information to inform annual changes in a state based land information dataset. They aim to provide a complete coverage of consistent land cover information for the state of Victoria which is updated on an annual basis. The Moderate Resolution Imaging Spectroradiometer (MODIS) MOD13Q1 product was used in conjunction with ancillary data such as mapped water bodies and land tenure to generate the maps. Vegetation indices such as Normalized Difference Vegetation Index (NDVI) and Enhanced Vegetation Index (EVI), which form part of the MOD13Q1 product, have been used extensively to map vegetation characteristics and land cover^10–13^. Characteristics of these indices can be used to identify land cover types based on growth patterns, which is useful for mapping crops^12,14,15^. The temporal frequency of MODIS image acquisition assists in identifying vegetation phenological features such as planting date, maturity onset and harvest date^13,16,17^. Programs including TIMESAT^18,19^, TiSeG^20^ and the Harmonic Analysis of Time-Series (HANTS)^21^ can be used to construct time-series of remotely sensed data, examine vegetation growth over a period of time and generate phenology parameters (such as the start and end of the vegetation growing season), and base and maximum levels of vegetation cover.

In order to extract accurate phenological characteristics time series data smoothing is used as without this, it is difficult to determine precise measurements of maximum vegetation cover and the rate and timing of increases in photosynthetic activity (vegetation ‘green up’)^22^. Many studies have employed smoothing techniques such as wavelet filters^6,17,23,24^, the harmonic analysis of NDVI time series program HANTS^5,25,26^, asymmetric Gaussian filter^27^, double-logistic filter^28^, and an adaptive Savitzky-Golay filter^13,29,30^. A Savitzky-Golay filter is used to produce the DLC product for Victoria. The methodology allows a land cover map to be regularly produced, providing consistent and current land cover information across the state. The land cover product uses a hierarchical classification scheme with three tiers. Decision trees, and rule sets derived using the C5.0 algorithm^31,32^, were used to classify annual DLC using a number of data inputs.

## Methods

### Land cover classification scheme

Annual DLC was produced as a pixel-based classification, with each 250 m pixel labelled with the dominant land cover for that year. The land cover classification scheme is a three-tiered hierarchy (primary, secondary and tertiary), as detailed in Table 1. The primary dominant land cover (PDLC) tier comprises three classes: water, woody vegetation and non-woody vegetation. The secondary dominant land cover (SDLC) tier divides the woody and non-woody vegetation classes into predominantly production, such as horticulture and agricultural production, and other vegetation such as native woody cover and pasture and grasslands. The tertiary dominant land cover (TDLC) tier further refines classifications of land cover types, particularly those associated with agricultural production.

### Data used

The land cover classifications are derived from a number of input data sources, summarized in Table 2 (available online only). The sources of data used, derivation of data and the work flow used to generate land cover classifications are detailed in the following sections.

#### Remotely sensed data

There are many satellite-based sensors which record spectral information about the earth’s surface such as Landsat, MODIS, SPOT, and Rapideye. They have varying spatial, temporal and spectral characteristics which make some more suitable for land cover mapping across a wide area than others.

Several alternative sensors were considered for this work. Data that was routinely acquired with minimal or no supply cost was considered to minimize the resources required to undertake this work, both at present and into the future. Landsat data, with a 30 m spatial resolution, are freely available, routinely acquired and are extensively used to map land cover^3,33–37^. However the temporal resolution of Landsat data are limited, with a revisit time of 16 days. Cloud cover often restricts the number of images available in areas such as south-western Victoria due to high cloud coverage. Reduced image availability due to cloud cover increases the difficulty of obtaining consistent multi-temporal acquisition across the 19 Landsat scenes required to achieve spatial coverage of Victoria. This presents limitations when discriminating land cover types, particularly when vegetation phenology derived from image time series are used or in agricultural landscapes where data availability during the growing season can be very low. During the development of this dataset, the security of data supply from the Landsat series of satellites was not guaranteed^35,38^, further limiting its suitability for this particular work.

Like Landsat data, MODIS imagery are also routinely acquired and are freely available. While the spatial resolution of MODIS imagery is coarser than Landsat data (250–1,000 m) the temporal frequency of the data are much improved compared with Landsat, which is useful for deriving vegetation phenology characteristics. MODIS data are also widely used in land cover mapping applications, particularly when maps covering a broad extent are required^10,13,39–42^. MODIS imagery have been used to map land cover on a per-pixel basis across areas such as sub-continents^41^, countries^42^, ecoregions^13^, sections of a country^6,15,40^, and globally^10,39^. The temporal frequency of the data are often a key factor used to distinguish land cover types using MODIS imagery^13,15,17,40^.

Previous studies have demonstrated that land cover mapping with good accuracy (73–79.3% (refs 10,^13^,^40^,^41^)) can be achieved on a per-pixel basis at a spatial resolution of 250–500 m using MODIS imagery. Land cover mapping utilizing data with a finer spatial resolution, such as Landsat, has been undertaken in Victoria, but at a smaller spatial extent rather than state-wide coverage, and these maps are not produced on a regular basis. Examples include the Strezleki Ranges (a 40 km^2^ study area in south-east Victoria)^7^, and the Katandra irrigation district (a 2000 ha study area in north-east Victoria)^2^. While the Landsat data may not provide the characteristics required for an annually updated land cover map with state-wide coverage, more localized data with information at a finer detail such as these could be used as Supplementary Information if required. This is beyond the scope of the work presented in this paper which presents the work undertaken to produce state-wide validated land cover maps from 2009 to 2013.

The MOD13Q1 Vegetation Indices product, which used for this work, has a swath of 2330 km, a spatial resolution of approximately 250 m and is produced as a composite image by selecting the highest quality pixel within a 16 day window, ensuring a cloud-free image is produced every 16 days. It includes spectral bands and indices particularly relevant to identifying vegetation characteristics; red and near-infrared (NIR) reflectance, NDVI, and EVI^43,44^.

Red reflectance responds to levels of chlorophyll in vegetation, while NIR reflectance is more sensitive to cell structure within leaves. NDVI has been shown to respond to levels of chlorophyll and vegetation biomass, although the response becomes saturated over areas of dense vegetation^43,45–48^. As a simple ratio, NDVI is also able to reduce topography effects on image data^49^. EVI is based on the soil adjusted vegetation index (SAVI)^50^, the atmospherically resistant vegetation index (ARVI) and the soil adjusted and atmospherically resistant vegetation index (SARVI)^51^. EVI is designed to be more sensitive to vegetation canopy structural features and in areas of high vegetative biomass, does not exhibit the same saturation issue as NDVI^43,46^. EVI reduces atmospheric and background reflectance effects^43^; however, it is more affected by topography than NDVI^52^. The responses of these data, and differences in response over time, help discriminate between different land cover types.

Red reflectance, NIR reflectance, NDVI and EVI datasets from the MOD13Q1 product were used to produce an annual DLC map. For each calendar year, an annual time series of 23 MOD13Q1 images^44^ were downloaded via the United States Geological Service (USGS) Reverb website (http://reverb.echo.nasa.gov/reverb). Images were clipped to the study area extent and formatted to generate input files for the TIMESAT program. The TIMESAT program^18,19^ was used to examine vegetation growth over a year and generate seasonal vegetation phenology parameters from EVI, NDVI, red and NIR reflectance MODIS data. An adaptive Savitzky-Golay filter was used in this study to smooth the image data, using a moving average^19^.

The seasonal parameters calculated were: the start and end of the growing season, the mid-point of the growing season, the length of the growing season, the base and maximum levels, the amplitude, the increase at the beginning of the season and decrease at the end of the season, and large and small integrals^18^. The annual standard deviation was calculated for the EVI, NDVI, red and NIR reflectance datasets separately. The processed images were reprojected from a Sinusoidal to VicGrid94 projection, maintaining the native pixel dimensions. The reprojected images were used to create a grid covering the state of Victoria, showing the MODIS pixel footprints across the study area. This pixel grid was used as a base for collecting ground data.

#### Ancillary data

A number of ancillary spatial data were also used to guide ground data collection and refine the land cover classifications. These were; Primary Production Landscapes, water bodies and parks and reserves: these data are maintained by the Victorian State Government (https://www.data.vic.gov.au/). Primary Production Landscapes are zones defined by features such as terrain, soil, broad climate aspects and land management^53^. Victoria is divided into six major Primary Production Landscape regions and 22 sub-regions. Geographic datasets of known water bodies^54^, including lakes, reservoirs, streams and wetlands, were used to determine the potential location of water. Land tenure for Victoria, which differentiates land in terms of ownership was also used^55^. Land tenure is recorded in many categories but for this project they were amalgamated to form four broad categories: private land, public land, parks and reserves (including National Parks and State Forests), and forestry plantations located on public land. These categories were used to guide the allocation of specific land cover classes on different types of land tenure (e.g., non-woody production was excluded from parks and reserves and forestry plantations located on public land, while woody production was excluded from parks and reserves but permitted in areas marked as forestry on public land).

#### Ground based data

**Figure 2:**
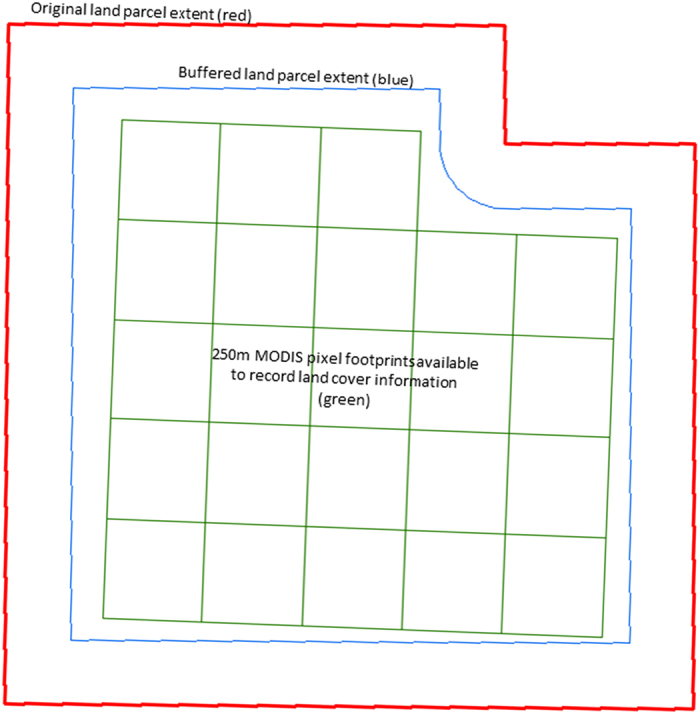
Example of field units used to collect ground data including a land parcel (red), a land parcel with a 150 m internal buffer applied (blue) and the 250 m MODIS pixels located within the buffered land parcel which could be used to record land cover information (green).

Ground-based data were collected for use in validation and calibration of the derived land cover maps. Ground-based data was collected within land parcels with a minimum area of 25 ha. An internal buffer of 150 m was applied to these parcels to limit the effects of boundaries and land cover transitions between parcels. Within these areas, MODIS 250 m pixel footprints were used as a minimum mapping unit, with final pixels used to record the land cover selected based on homogeneity and visibility^56,57^. The land cover present in one pixel can be influenced by surrounding pixels (spatial autocorrelation). This can affect the sample’s independence, which is an important consideration for validation and accuracy assessments of the classified product^58^. The influence of spatial autocorrelation was reduced in this study by using only one pixel per parcel. The pixel used was selected as the best representative of the particular parcel. Figure 2 shows a representation of the relationship between these field units. Ground data were collected in the field on a laptop computer using a geodatabase in ArcGIS^TM^ (ref. 57). For each pixel, the date, TDLC class, more detailed information about the cover present (such as specific crop type or forest canopy density) and an indication of information reliability (e.g., excellent or poor) was recorded. Ground data was collected for each year a land cover map was produced (2009–2013). Ground data was collected during Spring and early Summer (October to December each year) as agricultural crops reach maturity during this period and are more easily identified in the field.

A common approach to ground data collection is to use a random stratified clustered sampling approach, to provide a balance between a statistically rigorous sampling design and the practicalities and costs of ground data collection^58–60^. Victoria was stratified on the basis of six Primary Production Landscape regions. Within these regions, samples were clustered based on 1:25,000 topographical maps. Map sheets were randomly selected. Areas within a 20 km radius of the map sheet boundary were also included in the sampling structure to increase the number of ground data points collected.

As a guiding principle, other studies have suggested a minimum of 50 samples per class is required for validation purposes^58,61,62^. The number of samples collected per class is dependent on the resources available (time, money and personnel) and is also affected by the availability of accessible and suitable ground data sites for each class. This study aimed to collect a minimum of 400 samples per class, although some classes were consistently over- or under-sampled, dependent on their occurrence in the landscape. Half of the ground data collected each year were randomly selected and used as training data to derive classification rule sets. The remaining data were used to validate the land cover maps, with 50% of the validation data used to validate interim maps and 50% used to provide a final accuracy assessment of the land cover maps^63^.

### Producing an annual DLC product

**Figure 3:**
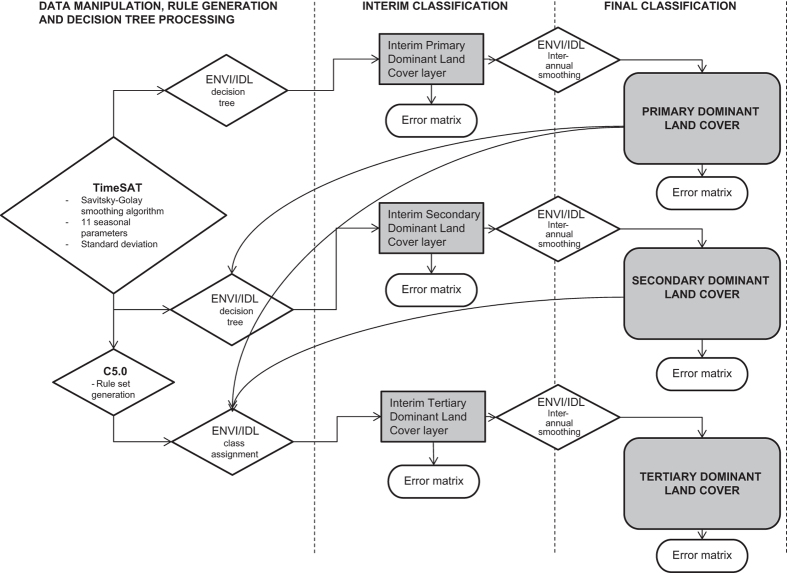
Schematic diagram of the process used to create an annual state-wide land cover map.

The annual DLC product was produced using a three-tiered hierarchical classification scheme. The land cover classifications are undertaken using the software ENVI 4.8^TM^ and IDL 8.0^TM^. Ground data and other ancillary data were compiled using ArcGIS^TM^. The PDLC and SDLC classifications were generated from decision trees with thresholds determined from ground data collected in 2012. The TDLC classification was generated from rule sets calculated using the See5^TM^ program, which uses the C5.0 algorithm^31,32^. The TDLC rule sets are specific for each year and are generated from the ground data and imagery collected during a single year. The broader classification layers (PDLC and SDLC) are also used when determining the final TDLC allocated to each pixel. Figure 3 provides a schematic diagram of the different processing steps used to generate the annual DLC product. The data used in each step are listed in Table 2 (available online only) which, together with Fig. 3 provides an overview of the dataset creation process.

#### Primary dominant land cover

A number of studies have utilized decision tree algorithms to generate land cover classifications from remotely sensed data^10,39,40^. Decision trees provide a robust, simple method of allocating data to classes based on value thresholds. They can incorporate categorical and non-parametric data, and thresholds can be defined without relying on calibration data^11^. A single decision tree was used to generate the PDLC classification for any given year to classify water, woody vegetation and non-woody vegetation. The decision tree was generated using the ENVI 4.8^TM^ and IDL 8.0^TM^ programs initially using thresholds based on observed values from ground data collected in 2012. This year was chosen as there were no major climatic events such as floods or widespread drought. Data from a single year was initially used to assess the performance of the decision tree when applied retrospectively. This was done to determine if the decision trees could be applied in years when ground data was not available (either historically or if ground data could not be collected in future years due to resourcing issues). PDLC and SDLC maps were calculated retrospectively from 2009–2011 as a result of this work and the 2013 maps were produced using the same decision tree. The accuracy of these maps is documented in the Technical Validation section and on the basis of these results no further calibration of the decision tree was undertaken.

Water was classified through an annual water mask generated using MODIS NDVI images for a calendar year and a map of known water bodies for the state of Victoria. The land cover map recorded the dominant, or majority, land cover over a given year. NDVI values were used to determine the presence and extent of water within known water bodies as water bodies do not always contain water, especially in drought years, and dry water bodies can sometimes be used for cropping or grazing. Water was considered present if a pixel was located within a mapped water body and had an NDVI value of <0.1 for more than 40% of the year (nine images in a year). This threshold was used to exclude intermittent surface water (such as flood waters) and non-permanent water bodies from the water class. The value was determined based on local knowledge and from testing a number of different thresholds to decide which best captured known water bodies that were covered by surface water for the majority of a given year.

This water mask was used for the primary, secondary and tertiary land cover classifications. The spatial data used in the decision tree to differentiate between non-woody and woody vegetation is listed in Table 2 (available online only). A error matrix was generated using the interim ground data validation set (Fig. 3). Inter-annual smoothing was used to create a final PDLC classification by restricting illogical land cover class transitions between years (for example, if a pixel was classified as woody vegetation in year one, non-woody vegetation in year two and woody vegetation in year three, then the pixel in year two was reclassified as non-woody vegetation). A error matrix was generated using the final validation data set. This, together with the smoothed classification layer, was used as the final PDLC classification product (Data Citation 1).

#### Secondary dominant land cover

The SDLC classification was generated by a second decision tree which separates woody vegetation cover into native woody cover and woody vegetation production (woody horticulture and plantation forestry), and separates non-woody vegetation cover into non-woody vegetation production (cropping or horticulture) and grasslands or pastures. The decision tree used the PDLC layer to determine the location of water and to restrict which SDLC classes could be attributed to a pixel. Spatial data used in the SDLC decision tree is listed in Table 2 (available online only). The process used to generate and validate the interim and final SDLC classification layers follows the method described for the PDLC classification layer. The final SDLC classification product and error matrix are available at Data Citation 1.

#### Tertiary dominant land cover

To produce the TDLC classification, the C5.0 algorithm in the See5^TM^ data mining program^31,32^ was used to generate a series of rules from ground-based training data which were collected annually (Fig. 3). C5.0, and its predecessor C4.5 construct decision trees based on observed patterns in the input data. The algorithm also breaks down the decision tree classifier into a series of rule sets, presenting the decision tree in a format which is more readily understood and interpreted, and often more accurate than the original decision tree^32^. Pruning was used to reduce errors introduced by over-fitting the data and to simplify the decision tree^32^. Decision tree algorithms such as C5.0 make no assumptions about the statistical distribution of the data, so are able to utilize non-parametric input data^64,65^. Each rule has a series of statements based on training data and input spatial layers used, and also has a confidence value (0–1) which is an indication of the prediction confidence associated with a rule^31^. The input spatial data used were the TIMESAT seasonal parameters and annual standard deviation derived from NDVI, EVI, red and NIR reflectance (Fig. 3 and Table 2 (available online only)). The layers used for different classes are selected by the algorithm and vary between years. Not all input layers were used in the final rule sets for each year.

The TDLC classification was produced using rules generated by the C5.0 algorithm and additional logical rules (for example, cereal crops could not be located on public land). Ancillary data including the PDLC and SDLC classifications, and land tenure, were used in this process. The rule set generated by C5.0 is not mutually exclusive and therefore multiple rules can produce a valid classification result for a single pixel. To determine the final class allocation for a given pixel, a class weighting was calculated based on the cumulative confidence values of all valid rules for each pixel within a single class^31^. The most likely and second most likely class for each pixel was determined. The final class was allocated based on a series of logical statements that considered the primary and secondary classes, and the most likely and second most likely tertiary class for each pixel. A error matrix was generated using the interim ground data validation set.

Inter-annual smoothing of the TDLC classifications to restrict illogical class transitions between years was done to produce a final TDLC classification, using a similar process to that used for the PDLC and SDLC classifications. A error matrix was generated using the final validation data set. This, and the smoothed classification layer were used as the final TDLC classification product (Data Citation 1).

## Data Records

Validated land cover maps which identified the dominant cover type on an annual basis at a broad-scale were produced from 2009 to 2013. The data are available as a raster data set (.dat files). For each year (2009–2013) three files are available: Primary Dominant Land Cover, Secondary Dominant Land Cover and Tertiary Dominant Land Cover. Accompanying the spatial data are a series of text files which contain detailed information regarding the accuracy assessment conducted for each classification file. These can be used to determine the appropriateness of the data for re-use by others. Both the data and metadata can be accessed at http://dx.doi.org/10.1594/PANGAEA.845372 (Data Citation 1).

The land cover maps are converted to vector format and merged with other land information (tenure and use) in the Victorian Land Use Information (VLUIS) product. The VLUIS datasets are available as a biennial product for the following years: 2006/07, 2008/09, 2010/11, 2012/13. The 2006/07 and 2008/09 VLUIS datasets include SDLC maps for 2006, 2007 and 2008 which have no accuracy associated with them as there is no ground data available to provide validation^66^. A lack of ground data also meant that TDLC maps for these three years are not available. The VLUIS dataset can be accessed through the Dryad Digital Repository (Data Citation 2) or by searching for the term ‘VLUIS’ on the Victorian Government websites Data.Vic^67^ and Victorian Resources Online^9^.

The methods outlined in this paper are intended to be used to create these annual land cover maps into the future, with data made available as it is finalized. Future updates will be made available on the Victorian Government websites Data.Vic^67^ and Victorian Resources Online^9^ as part of the VLUIS dataset.

## Technical Validation

### Land cover maps 2009–2013

While the production of a single land cover map is a useful reference, regularly updated land cover maps are required to document changes through time^13,42^, and observe differences in land cover types which change frequently, such as crop rotations between cereals, brassicas, legumes, pastures and grasslands and bare ground. Annual DLC maps were produced from 2009 to 2013, with comparable accuracy between years at each level of detail. Error matrices were generated for each map (PDLC, SDLC and TDLC for each year) using independent sets of ground validation data. The total number of ground data points assessed each year varied dependent on resources available to collect them. The number of ground data points assessed was 4681 in 2009, 4989 in 2010, 4470 in 2011, 3767 in 2012, and 6034 in 2013.

The overall accuracy for each map (based on a final accuracy assessment using an independent 25% random selection of ground data) is presented in Table 3. The PDLC maps had an accuracy of >90% for all years. The overall accuracy of the SDLC maps ranged from 72 to 80%. The TDLC maps had an accuracy of between 61 and 68%. The primary level classification consists of three classes: water, non-woody vegetation and woody vegetation. At this level of class detail, most MODIS pixels are predominantly one of these three classes. This is particularly true across public land and in agricultural areas, which account for 38 and 55% of the state respectively. The heterogeneity of land cover types at the spatial scale of a MODIS pixel increases as the detail of land cover classes increases, as occurs in the secondary and tertiary land cover classes used in this study. The influence of this increase in heterogeneity is reflected in the lower accuracy of the secondary and tertiary level maps compared with the primary level maps. While the accuracy and level of class detail in the maps is influenced somewhat by the heterogeneity of the landscape, they still provide consistent, repeatable information about land cover at a broad scale which is important for many applications.

### PDLC maps 2009–2013

The PDLC level gives a broad overview of the state in terms of woody and non-woody vegetation. It also identifies water bodies, and the extent of water in them. A standard decision tree was used to produce all PDLC classifications from 2009 to 2013, which have overall classification accuracies greater than 91% (Table 3).

**Figure 4:**
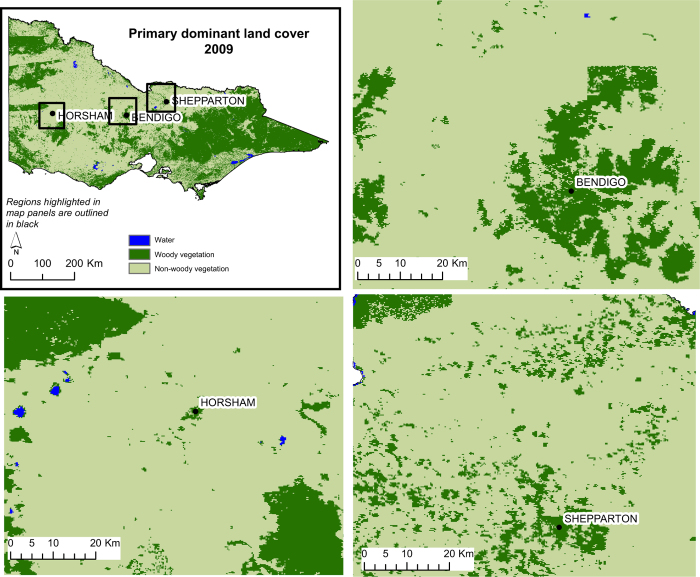
Primary dominant land cover for 2009 at a state level and for three smaller regions.

Table 4 shows the overall, and class specific, producer and user accuracies for the PDLC classification layer from 2009, where all classes had a producer and user accuracy >84%. Example maps of the 2009 PDLC are shown in Fig. 4. The 2009 PDLC classification identified water very accurately, but misclassified some woody vegetation as non-woody vegetation. In 2013 and 2010, errors in classifying water were an issue: for the 2010 PDLC classification, the producer accuracy for water was 69% while in 2013 it was 34.6%. In these cases, water was misclassified as non-woody vegetation. All other accuracy estimates for PDLC classifications from 2009 to 2013 were >80%.

### SDLC maps 2009–2013

The SDLC maps divide the woody and non-woody vegetation classes from the PDLC maps into production classes (woody and non-woody production) and cover associated with less intensive uses (native woody cover and pastures and grasslands). A single decision tree was also used to produce all SDLC classifications from 2009 to 2013, which had overall classification accuracies post inter-annual smoothing ranging from 73 to 81% (Table 3).

**Figure 5:**
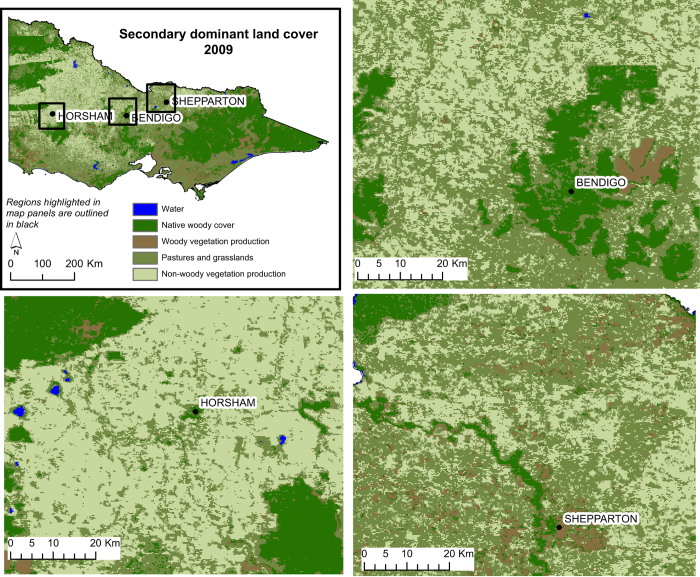
Secondary dominant land cover for 2009 at a state level and for three smaller regions.

Table 5 shows the overall, and class producer and user accuracy for the 2009 SDLC classification layer. Example maps of the 2009 SDLC are shown in Fig. 5. The water classification is taken from the PDLC classification layer. In 2009, errors occurred as misclassification between pastures and grasslands and non-woody vegetation production; where approximately 40% of pastures and grasslands were misclassified as non-woody production and 15% of non-woody production was misclassified as pasture and grasslands. Native woody cover was misclassified as woody vegetation production, while woody vegetation production was misclassified as pasture and grasslands as well as native woody cover (Table 6).

The user accuracy for pastures and grasslands in 2010 was <40%, with most errors occurring as non-woody vegetation production misclassified as pastures and grasslands. The accuracy assessments for the remaining classes in 2010 were >68%. In 2011, the user accuracy for pastures and grasslands and native woody cover, and the producer accuracy for non-woody vegetation production were <65%, with all other class accuracy assessments >75%. In 2012, pastures and grasslands were frequently misclassified as non-woody vegetation production. Misclassification also occurred between woody vegetation production and native woody cover.

Pastures and grasslands in 2012 had a producer and user accuracy <52%, while the user accuracy for native woody cover and producer accuracy for woody vegetation production was <60%. All other class accuracy estimates were >80%. In the 2013 SDLC map, pastures and grasslands misclassified as non-woody vegetation production accounted for the majority of errors.

PDLC and SDLC maps were produced using single decision trees where the thresholds used to distinguish cover types were determined based on ground data collected in 2012 and subsequently not altered between years. As such, they required no annual calibration with independent ground data. Ground data were used solely to validate the resulting classifications, which were produced with consistent levels of accuracy between years (Table 3).

**Figure 6:**
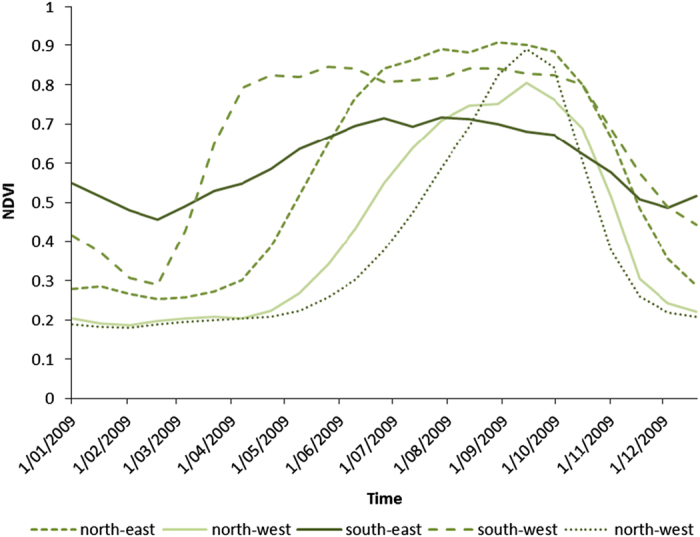
Representative pixel-wise annual time series of smoothed NDVI (MOD13Q1) for pastures and grasslands in 2009, selected from north-east, north-west, south-east and south-west regions of Victoria.

The approach used was able to accommodate the variable climatic conditions both between regions and between years from 2009 to 2013, which had significant impacts on vegetation growth patterns and agricultural production across the state. There are substantial differences in climatic conditions across different regions of Victoria. This diversity affects not only the type of land cover across the state, but typical growth patterns within the same land cover. For example, Fig. 6 shows a selection of annual growth patterns for pasture located in different regions of Victoria. Pasture growth in the south-east is generally consistent throughout the year because of the amount of rainfall the area receives. The south-east climate is generally wetter throughout the entire year (e.g., Warragul in the south-east has an average annual rainfall of 1,022 mm compared with Horsham in the west which has an average annual rainfall of 260 mm). Pasture growth in other areas of the state (e.g., the north-east and south-west) are more influenced by seasonal rainfall over autumn and winter (March—November) or irrigation water being used to supplement rainfall over spring and summer (September—November). These differences in climate and pasture growth patterns result in the differences seen in Fig. 6, but the approach used to generate PDLC and SDLC maps was able to account for this variation with reasonable accuracy.

Climatic conditions vary not only across different regions in Victoria, but between the years when these maps were produced. While the beginning of 2009 was very hot and dry, some areas, including the north-west and western regions experienced average annual, while central and eastern regions recorded below average annual rainfall^68^. In contrast, 2011 recorded higher than average rainfall across the state, and was the 12th wettest year recorded^69^. Widespread flooding was recorded in the early part of 2011 across the state; however, the central and northern regions experienced a drier than average winter, while some south-eastern regions recorded average or wetter than average rainfall^69^. The timing, as well as location, of rainfall impacts vegetation growth, especially in dryland cropping regions. 2013 recorded variable, but near average, rainfall across the state, with the north-west of the state experiencing drier conditions than coastal areas^70^.

A single set of thresholds were used to derive PDLC and SDLC (one set of thresholds for each) for 2009–2013. Similar accuracy was achieved for each of the years using these common thresholds despite climatic conditions varying considerably between years, producing a reliable multi-year land cover data set at a broad spatial scale. This indicates the thresholds used do not require recalibration each year to account for the influence on varying climatic conditions on vegetation growth patterns. As the thresholds are not greatly influenced by inter-annual climatic variations and are robust enough to be used between years without recalibration, they could be used to generate PDLC and SDLC maps for years where ground data has not been collected and cannot be assembled retrospectively (e.g., 2000–2008) and a broad-scale estimate of land cover is required. This has been done for 2006, 2007 and 2008 where estimated land cover information (PDLC and SDLC) was required for inclusion in the Victorian Land Use Information System (VLUIS)^66^. While a lack of ground data prohibits an accuracy assessment of the maps, a similar accuracy to those produced from 2009–2013 could be used as an estimate of accuracy.

### TDLC maps 2009–2013

**Figure 7:**
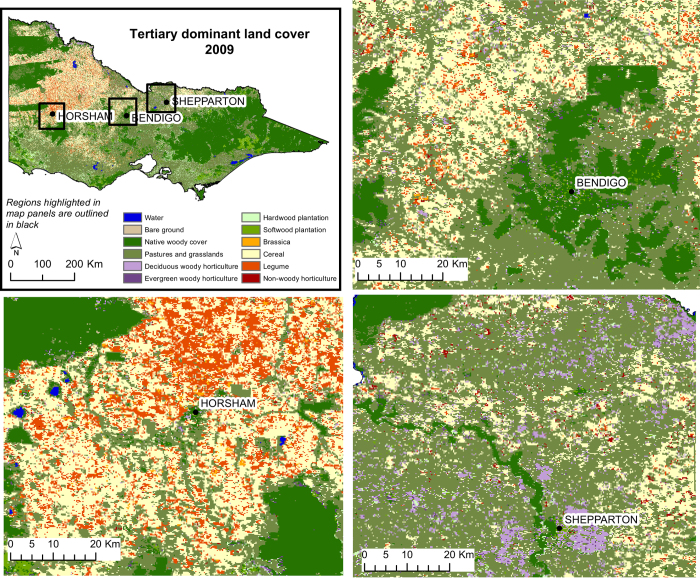
Tertiary dominant land cover for 2009 at a state level and for three smaller regions.

The TDLC classification layer was produced using a rule set derived from training data interpreted by the C5.0 algorithm, which can process non-parametric data. The accuracy of the TDLC classifications ranged from 61.6 to 68.3% (Table 3). Example maps of the 2009 TDLC classification are shown in Fig. 7.

Producer and user accuracies for the 12 TDLC classes from the 2009 TDLC map are given in Table 7 (available online only). Classes are distinguished based on growth patterns occurring over one year (23 MODIS images). Seasonal parameters generated in TIMESAT are able to distinguish between different land cover classes at a coarse spatial resolution, although some are more easily separable than others. Table 8 (available online only) provides a summary of the most and least accurately identified land cover classes. Detailed error matrices for each year are distributed with the land cover maps (Data Citation 1).

Water and woody vegetation classes (native woody cover, softwood and hardwood plantations, and deciduous woody cover) generally had high accuracy across all years, with accuracies greater than 71%, except for native woody cover (user accuracy of 64.1%). Tertiary land cover classes that were consistently difficult to characterize included bare ground and non-photosynthetic material, non-woody horticulture (e.g., tomatoes and potatoes) and evergreen woody horticulture (e.g., olive and citrus trees) (Table 8 (available online only)). Factors contributing to the misclassification of pixels include: the similarity of annual vegetation growth patterns between some classes, limited spectral information and the spatial resolution of the satellite imagery (resulting in pixels containing multiple land cover types). Land cover types, such as non-woody horticulture, can occur in areas smaller than the spatial resolution of the MODIS imagery used. These land cover types may benefit from mapping undertaken at a finer spatial resolution using alternative sensors, which, while beyond the scope of the work presented here, could be explored through future work in this area. Some classes, such as pastures and grasslands, showed a varied spectral response, both over time and between training samples (Fig. 4). This may be due to a wide range of factors including geographical location, the inclusion of different species in one class (native and exotic plant species), annual and perennial plants incorporated in the one class, and different land management options (such as grazing schedules and irrigation). These characteristics make some classes more difficult to distinguish than others.

Table 9 (available online only) presents a more detailed error matrix for the 2009 TDLC classification. These detailed error matrices are available for all maps and are distributed with the data (Data Citation 1). Woody vegetation classes were more reliably identified than non-woody vegetation classes. While variations in land cover class definitions make it difficult to compare other studies directly, some have also shown higher accuracies when identifying woody vegetation classes compared with other land cover classes^3,41^. This trend may be due in part to lower annual temporal and spatial variation in growth characteristics for woody vegetation classes compared with non-woody vegetation classes such as cereals, brassicas and legumes.

## Usage Notes

The validated land cover maps produced from 2009 to 2013 are intended as a source of broad land cover information, providing a complete coverage of land cover information for the state of Victoria which is updated on an annual basis. The products aim to provide a consistent, repeatable source of land cover information that can be reproduced on an annual basis in a resource efficient manner. The 250 m gridded land cover maps can be used as a stand-alone products, with users selecting which level of detail they require (primary, secondary or tertiary) for their particular purpose. Some classes, such as grasslands and pastures, and some non-woody agricultural classes have higher error rates than classes such as water and woody vegetation. Data accuracy is an important consideration for users when determining the suitability of the data for a particular purpose. Detailed error matrices (such as that shown in Table 9 (available online only)) are available for each map, and are distributed with the datasets (Data Citation 1). These are intended for interrogation by the user to determine if the data suit their requirements.

The classifications are also integrated within the VLUIS product, which provides an integrated spatial database of land tenure, land use and land cover information for each land parcel in Victoria^4,66^. Land parcels smaller than 12.5 ha are not attributed with a land cover class due to the coarse 250 m spatial resolution of the MODIS imagery^66^. The VLUIS provides a fundamental dataset which can be manipulated for a variety of purposes such as analyzing the spatial distribution of industries, documenting the areal and spatial change in annual crop plantings, responses of land cover to climate conditions (such as drought), and long-term land cover change and trends.

## Additional Information

**How to cite this article:** Sheffield, K. *et al.* Mapping dominant annual land cover from 2009 to 2013 across Victoria, Australia using satellite imagery. *Sci. Data* 2:150069 doi: 10.1038/sdata.2015.69 (2015).

## Supplementary Material



## Figures and Tables

**Table 1:** Primary, secondary and tertiary dominant land cover classes.

**Primary dominant land cover classes**	**Secondary dominant land cover classes**	**Tertiary dominant land cover classes**
Water	Water	Water
Woody vegetation	Native Woody Cover	Native Woody Cover
	Woody vegetation—production	Deciduous woody horticulture
		Evergreen woody horticulture
		Hardwood plantation
		Softwood plantation
Non-woody vegetation	Pasture and grassland	Pasture and grassland
	Non-woody vegetation—production	Brassicas
		Cereals
		Legumes
		Non-woody horticulture
		Bare ground and non-photosynthetic vegetation

**Table 2:** Spatial data used to generate Primary, Secondary and Tertiary Dominant Land Cover classifications (specific data references are cited in the text)

**Input data**	**Variable**	**Source**	**Temporal resolution**	**Dataset generation**
MOD13Q1	NDVI annual time series	Derived from MODIS imagery supplied by NASA	Calculated annually	PDLC
	NDVI annual standard deviation	Derived from MODIS imagery supplied by NASA	Calculated annually	PDLC, SDLC, TDLC
	Red reflectance annual standard deviation	Derived from MODIS imagery supplied by NASA	Calculated annually	PDLC, TDLC
	NIR reflectance annual standard deviation	Derived from MODIS imagery supplied by NASA	Calculated annually	SDLC, TDLC
	EVI annual standard deviation	Derived from MODIS imagery supplied by NASA	Calculated annually	TDLC
	NDVI amplitude, base, start of the season, end of the season, large integral, left derivative, right derivative, maximum, small integral, mid-point of the season	Derived from MODIS imagery supplied by NASA using TIMESAT software	Calculated annually	PDLC (amplitude, base and large integral only), SDLC (amplitude and base only), TDLC (all)
	EVI amplitude, base, start of the season, end of the season, large integral, left derivative, right derivative, maximum, small integral, mid-point of the season	Derived from MODIS imagery supplied by NASA using TIMESAT software	Calculated annually	TDLC
	NIR amplitude, base, start of the season, end of the season, large integral, left derivative, right derivative, maximum, small integral, mid-point of the season	Derived from MODIS imagery supplied by NASA using TIMESAT software	Calculated annually	SDLC (base only), TDLC (all)
	Red reflectance amplitude, base, start of the season, end of the season, large integral, left derivative, right derivative, maximum, small integral, mid-point of the season	Derived from MODIS imagery supplied by NASA using TIMESAT software	Calculated annually	PDLC (base only), SDLC (amplitude only), TDLC (all)
Primary Production Landscapes	Primary Production classes	Victorian State Government	Updated as required	PDLC, SDLC, TDLC
Land tenure	Broad land tenure classes	Victorian State Government	Updated annually	PDLC, SDLC, TDLC
Known hydrological features	Mapped hydrological features (polygons)	Victorian State Government	Updated annually	PDLC
SDLC	SDLC classes	Victorian State Government	Updated annually	TDLC
PDLC	PDLC classes	Victorian State Government	Updated annually	SDLC, TDLC
Ground data	TDLC class, assessed on-ground corresponding to the footprint of one MODIS pixel	Victorian State Government	Updated annually	TDLC

**Table 3:** Summary of overall final classification accuracies (%) for primary, secondary and tertiary dominant land cover classifications for 2009–2013.

	**2009**	**2010**	**2011**	**2012**	**2013**
Primary dominant land cover	91.0%	91.0%	94.3%	96.8%	92.9%
Secondary dominant land cover	72.9%	77.4%	74.5%	80.9%	76.3%
Tertiary dominant land cover	68.3%	64.5%	64.5%	61.6%	66.1%

**Table 4:** Error matrix summary for the 2009 primary dominant land cover classification

	**Producer accuracy**	**User accuracy**
Water	98.4%	100.0%
Woody vegetation	85.3%	97.0%
Non-woody vegetation	96.4%	84.3%
Overall accuracy: 91.0%.		

**Table 5:** Error matrix summary for the 2009 secondary dominant land cover classification

	**Producer accuracy**	**User accuracy**
Water	98.4%	100.0%
Native woody cover	61.3%	51.7%
Woody vegetation production	67.6%	86.2%
Pastures and grasslands	53.2%	39.3%
Non-woody production	83.5%	79.9%
Overall accuracy: 72.9%.		

**Table 6:** Detailed error matrix for the 2009 secondary dominant land cover classification

	**Water**	**NWC**	**WVP**	**PGR**	**NWP**
Unknown	0.0%	7.3%	0.0%	0.0%	0.0%
Water	98.4%	0.0%	0.0	0.0%	0.0%
NWC	0.0%	61.3%	16.6%	1.6%	1.1%
WVP	0.0%	27.4%	67.6%	6.5%	0.4%
PGR	1.6%	4.0%	13.8%	53.2%	15.0%
NWP	0.0%	0.0%	2.0%	38.7%	83.5%
NWC, Native woody cover; NWP, Non-woody production; PGR, Pastures and grasslands; WVP, Woody vegetation production.					
					

**Table 7:** Error matrix summary for the 2009 tertiary dominant land cover classification

	**Producer accuracy**	**User accuracy**
Water	98.4%	100.0%
Bare ground and non-photosynthetic vegetation	24.2%	85.7%
Native woody cover	80.7%	64.1%
Pastures and grasslands	63.7%	47.9%
Deciduous woody horticulture	75.8%	92.2%
Evergreen woody horticulture	57.7%	68.2%
Hardwood plantations	73.4%	76.5%
Softwood plantations	71.8%	75.4%
Brassica	28.6%	50.0%
Cereal	77.4%	53.3%
Legume	64.0%	59.7%
Non-woody horticulture	5.9%	33.3%
Overall accuracy: 68.3%.		

**Table 8:** Summary of most and least accurately classified tertiary dominant land cover classes 2009–2013

**TDLC**	**User accuracy notes**	**Producer accuracy notes**
**Most accurately classified classes 2009–2013**		
Water	User accuracy >94% 2009–2013	Producer accuracy >80% except in 2013 (34.6%) and 2010 (69.4%)
Native woody cover	User accuracy >75% in 2010 and 2011, 67.5% in 2012 and <65% in 2013 and 2009	Producer accuracy >80% except in 2013 (69.5%)
Deciduous woody horticulture	User accuracy >74% 2009–2013	Producer accuracy >75% 2009–2013
Hardwood plantations	User accuracy >72% except in 2012 (63.2%)	Producer accuracy >66% except in 2012 (38.7%)
Softwood plantations	User accuracy >75% except for 2012 (68.8%) and 2013 (70.6%)	Producer accuracy >70% except in 2012 (48.7%)
		
**Least accurately classified classes 2009–2013**		
Brassicas	User accuracy <62% 2009–2013	Producer accuracy <61% 2009–2013
Cereals	User accuracy <65% 2009–2013	Variable producer accuracy; >83% in 2013 and 2012, 77.4% in 2009, and <47% in 2010 and 2011
Non-woody horticulture	User accuracy <33% except for 2011 (92.3%) and 2012 (80%)	Producer accuracy <40% 2009–2013
Evergreen woody horticulture	Variable user accuracy; <70% in 2009, 2011 and 2013, 77.8% in 2010 and 85.7% in 2012	Producer accuracy <58% 2009–2013
Bare or non-photosynthetic vegetation	User accuracy <66% except in 2012 (80%) and 2009 (85.7%)	Producer accuracy <25% 2009–2012

**Table 9:** Detailed error matrix for the 2009 tertiary dominant land cover classification

	**WAT**	**BNP**	**NWC**	**PGR**	**DWH**	**EWH**	**HPL**	**SPL**	**BRA**	**CER**	**LEG**	**NWH**
**UNK**	0.0%	0.0%	7.3%	0.8%	0.0%	0.0%	0.0%	0.8%	0.0%	0.0%	0.0%	0.0%
**WAT**	**98.4%**	0.0%	0.0%	0.0%	0.0%	0.0%	0.0%	0.0%	0.0%	0.0%	0.0%	0.0%
**BNP**	0.0%	**24.2%**	0.0%	0.8%	0.0%	0.0%	0.0%	0.0%	0.0%	0.0%	1.8%	11.8%
**NWC**	0.0%	32.3%	**80.7%**	0.8%	5.7%	7.7%	0.0%	2.4%	0.0%	0.0%	0.0%	17.7%
**PGR**	1.6%	12.9%	3.2%	**63.7%**	11.3%	7.7%	12.9%	9.7%	21.4%	4.0%	3.6%	47.1%
**DWH**	0.0%	0.8%	0.0%	0.0%	**75.8%**	23.1%	0.0%	0.0%	0.0%	0.0%	0.0%	5.9%
**EWH**	0.0%	0.0%	0.8%	0.0%	4.0%	**57.7%**	0.0%	0.8%	0.0%	0.0%	0.0%	0.0%
**HPL**	0.0%	0.0%	1.6%	4.8%	0.8%	3.8%	**73.4%**	14.5%	0.0%	0.0%	0.0%	0.0%
**SPL**	0.0%	0.0%	6.4%	2.4%	1.6%	0.0%	12.9%	**71.8%**	0.0%	0.0%	0.0%	0.0%
**BRA**	0.0%	0.8%	0.0%	0.0%	0.0%	0.0%	0.0%	0.0%	**28.6%**	1.6%	0.9%	0.0%
**CER**	0.0%	12.9%	0.0%	23.4%	0.0%	0.0%	0.8%	0.0%	21.4%	**77.4%**	29.7%	11.8%
**LEG**	0.0%	16.1%	0.0%	2.4%	0.8%	0.0%	0.0%	0.0%	28.6%	16.1%	**64.0%**	0.0%
**NWH**	0.0%	0.0%	0.0%	0.8%	0.0%	0.0%	0.0%	0.0%	0.0%	0.8%	0.0%	**5.9%**
UNK=Unknown.												
WAT=Water.												
BNP=Bare and non-photosynthetic material.												
NWC=Native woody cover.												
PGR=Pastures and grasslands.												
DWH=Deciduous woody horticulture.												
EWH=Evergreen woody horticulture.												
HPL=Hardwood plantations.												
SPL=Softwood plantations.												
BRA=Brassicas.												
CER=Cereals.												
LEG=Legumes.												
NWH=Non-woody horticulture.												
